# The locus coeruleus input to the rostral ventromedial medulla mediates stress-induced colorectal visceral pain

**DOI:** 10.1186/s40478-023-01537-6

**Published:** 2023-04-17

**Authors:** Dexu Kong, Yunchun Zhang, Po Gao, Chao Pan, Haoyue Deng, Saihong Xu, Dan Tang, Jie Xiao, Yingfu Jiao, Weifeng Yu, Daxiang Wen

**Affiliations:** 1 Department of Anesthesiology, Renji Hospital, Shanghai Jiaotong University School of Medicine, Shanghai, 200127 China; 2 Key Laboratory of Anesthesiology (Shanghai Jiao Tong University), Ministry of Education, Shanghai, China

**Keywords:** Pathological stress, Colorectal visceral pain, Locus coeruleus, Rostral ventromedial medulla, Neural circuit

## Abstract

Unlike physiological stress, which carries survival value, pathological stress is widespread in modern society and acts as a main risk factor for visceral pain. As the main stress-responsive nucleus in the brain, the locus coeruleus (LC) has been previously shown to drive pain alleviation through direct descending projections to the spinal cord, but whether and how the LC mediates pathological stress-induced visceral pain remains unclear. Here, we identified a direct circuit projection from LC noradrenergic neurons to the rostral ventromedial medulla (RVM), an integral relay of the central descending pain modulation system. Furthermore, the chemogenetic activation of the LC-RVM circuit was found to significantly induce colorectal visceral hyperalgesia and anxiety-related psychiatric disorders in naïve mice. In a dextran sulfate sodium (DSS)-induced visceral pain model, the mice also presented colorectal visceral hypersensitivity and anxiety-related psychiatric disorders, which were associated with increased activity of the LC-RVM circuit; LC-RVM circuit inhibition markedly alleviated these symptoms. Furthermore, the chronic restraint stress (CRS) model precipitates anxiety-related psychiatric disorders and induces colorectal visceral hyperalgesia, which is referred to as pathological stress-induced hyperalgesia, and inhibiting the LC-RVM circuit attenuates the severity of colorectal visceral pain. Overall, the present study clearly demonstrated that the LC-RVM circuit could be critical for the comorbidity of colorectal visceral pain and stress-related psychiatric disorders. Both visceral inflammation and psychological stress can activate LC noradrenergic neurons, which promote the severity of colorectal visceral hyperalgesia through this LC-RVM circuit.

## Introduction

Pathological stress cannot be avoided in the present competitive world. Unlike physiological stress, which carries survival value, pathological stress is associated with detrimental effects on physical health and the occurrence of disease [21]. Pathological stress is often comorbid with alterations in gastrointestinal tract function, which in turn lead to visceral hyperalgesia [6, 30, 43, 49, 68]. Psychological and pharmacological interventions that mediate stress perception have been demonstrated to ameliorate visceral pain symptoms [57, 62]. In addition, visceral pain and stress-related psychiatric disorders exhibit high comorbidity [12, 77], which tends to create a positive link between stress-related psychiatric disorders and visceral pain that is difficult to decouple when encountered in many acute and chronic clinical settings [76]. The mechanisms of this association warrant more research attention.

As the major producer of noradrenaline (NA) in the central nervous system, the locus coeruleus (LC) sends widespread projections throughout the central nervous system and acts as the main stress-responsive nucleus implicated in mediating psychiatric disorders [45, 63, 69]. Because the chemogenetic blockade of the LC-basolateral amygdala circuit can reverse pain-induced psychiatric disorders but has no effect on the emergence of sensory hyperalgesia [41], the LC may contribute to pain and stress-related psychiatric disorders through distinct neural circuits. In addition, previous findings suggested that stress elicits visceral hyperalgesia and the functional reorganization of pain circuits, including functional activation of the LC [1, 37, 38, 42, 52, 61]; however, responses to colorectal distention were inhibited during coeruleospinal (LC/SC) electrical stimulation [36, 37]. These results suggest that the LC may mediate stress-induced visceral hyperalgesia through other neural circuits rather than the classical descending LC/SC projection.

The rostral ventromedial medulla (RVM) is a key relay in the descending pain modulation system [13, 20]. Electrical or chemical stimulation of the RVM produces either descending inhibition or pain by regulating spinal dorsal neuron responses and spinal nociceptive reflexes to noxious stimuli [10, 48, 51, 65]. In terms of visceral pain, several studies have implicated a major role for the RVM in central processing of visceral pain, such as urinary bladder distention [54], pancreatic pain [73], colorectal distention and visceral hyperalgesia induced by chemical intracolonic irritants [35, 78]. Furthermore, previous studies have observed functional connectivity between the RVM and LC by using resting-state functional magnetic resonance imaging and fiber photometry systems. Specifically, changes in neuronal responses in the RVM and LC to stress stimuli or visceral nociceptive stimuli were identified [28, 29, 47, 50]. Overall, these findings suggest that the LC-RVM circuit may be a convergence point between stress and colorectal visceral pain, but the detailed neural circuit and cause-and-effect relationship between the LC and RVM in stress-induced visceral pain remain far from clear.

In this study, we used retrograde labeling and projection-specific chemogenetic manipulation of the LC-RVM circuit in mice to determine the precise role of the LC-RVM circuit in stress-induced effects on visceral pain. To this end, a model of the comorbidity of stress and colorectal visceral pain induced by dextran sulfate sodium (DSS) and chronic restraint stress (CRS) was used.

## Materials and methods

### Animals

Naïve male C57BL/c mice obtained from the vivarium of Shanghai Jiaotong University School of Medicine were used in all experiments. Animals weighed 22–26 g (6–8 weeks of age) and were housed in a temperature-controlled room (22–25 °C) illuminated from 07:00 to 19:00. Food and water were available ad libitum. All animal care and experimental procedures followed the Guiding Principles on the Care and Use of Animals and the Animal Management Rule of the Ministry of Public Health, People's Republic of China (documentation 545, 2001) and were approved by the Ethnic Committee for Experimental Use of Animals of Shanghai Jiaotong University School of Medicine.

### Stereotaxic injection

Cre-dependent adeno-associated viruses (AAVs) were used to manipulate neural activity in the LC in this study. AAV injections and cranial window implantations were performed as previously described [15, 59]. Mice were deeply anesthetized with pentobarbital sodium by intraperitoneal injection (i.p.) (0.1 g/kg) and positioned in a stereotaxic apparatus (RWD, Shenzhen, China). The skull was exposed via a small incision, and three small holes were drilled (0.50 mm drill bit) into the skull to introduce a microinjection glass pipette into bilateral LC (AP, −5.40 mm, ML, ± 0.80 mm, DV, 3.80 mm) in a volume of 150 nL for each side at 15 nL/min and into the RVM (AP,  − 5.70 mm, ML, 0.00 mm, DV, 5.80 mm) in a volume of 300 nL at 30 nL/min. The syringe was slowly retracted after an additional 10 min diffusion of the virus. The skin was sutured, and the animals were allowed to recover in prewarmed cages before returning to the home cage. Postoperative antibiotic therapy was administered in the form of ceftriaxone sodium 3 days after surgery (i.p.) (0.1 g/kg/day, once a day).

For LC→RVM circuit tracing, a dual viral approach was performed to label the LC neurons projecting to the RVM: bilateral injection of AAV2/9-CAG-DIO-mCherry-WPRE (~ 1.1 × 10^12^ vg/mL) targeting the LC and of AAV2/retro-CMV-Cre-WPRE (~ 2.00 × 10^12^ vg/mL) targeting the RVM. To selectively label the NA^LC^→RVM circuit, AAV2/9-CAG-DIO-EGFP-WPRE (~ 2.0 × 10^12^ vg/mL) was bilaterally injected into the LC and AAV2/retro-TH-Cre (~ 2.00 × 10^12^ vg/mL) into the RVM.

For chemogenetic manipulation, AAV2/9-CAG-DIO-hM3D(Gq)-mCherry (hM3Dq-mCh) (~ 2.00 × 10^12^ vg/mL) or AAV2/9-CAG-DIO-hM4D(Gi)-mCherry (hM4Di-mCh) (~ 2.00 × 10^12^ vg/mL) was bilaterally injected into the LC, and AAV2/9-CAG-DIO-mCherry (mCh) (~ 2.00 × 10^12^ vg/mL) was used as the control together with AAV2/retro-TH-Cre (~ 2.00 × 10^12^ vg/mL) injection into the RVM. Mice were used 3 weeks after AAV injection. All viruses were purchased from BrainVTA (Wuhan, China).

### Chemogenetic manipulation

For the chemogenetic manipulation of the NA^LC^→RVM circuit, mice were injected (i.p.) (0.03 mg/kg dissolved in normal saline) for activation or (i.p.) (0.1 mg/kg dissolved in normal saline) for inhibition with clozapine 30 min before behavioral assessment, unless otherwise stated. Controls received an equivalent volume of saline. This dosage of clozapine was selected based on published studies [22].

### Dextran sulfate sodium administration

To induce DSS colitis, mice were offered filter-sterilized 2.5% (w/v) DSS (Mpbio, Canada) as their sole drinking water for 7 days ad libitum [18]. Mice were monitored, and the disease activity index score was determined as described previously, including assessments of diarrhea, rectal bleeding, and initial weight loss. Scores were defined as follows: diarrhea score 0 (normal), 2 (soft), or 4 (watery stool), and rectal bleeding score 0 (no blood), 2 (visual pellet bleeding), or 4 (gross bleeding, blood around anus) [33]. Weight loss was measured as a percentage relative to the initial weight of each individual. The distal colons were dissected after DSS treatment (Day 7) and compared with those of the control group rats that received only normal drinking water throughout the experimental period.

### Restraint stress

The restraint stress model was used to study psychological stress [16]. Restraint stress was performed as described previously [60]. Briefly, mice in the chronic restraint stress (CRS) group were immobilized for 2 hours daily for 14 consecutive days in 50 mL conical tubes with holes to allow the mice to breath. Mice in the acute restraint stress (ARS) group were immobilized once for 2 h. The control group (Con) was allowed to freely move in the cage without access to water or food. In addition, the CRS combined with 1% DSS model was used to replicate the common condition that psychological stress predispose the individual to develop visceral hyperalgesia.

### Abdominal mechanical sensitivity

The abdominal mechanical sensitivity of mice was measured using abdominal withdrawal reflex (AWR) tests as described previously [3, 35]. Briefly, mice were habituated to a metallic mesh floor covered with plastic boxes for 1 h daily for 3 days prior to testing, and the abdomen was shaved 1 day before experiments. The abdominal area was stimulated with calibrated von Frey filaments (VFFs) of different applied forces (subthreshold mechanical stimuli (“allodynia” corresponds to 0.07 g force application) and painful stimuli (“hyperalgesia” thicker filaments, correspond to 0.16 and 1 g force application)) 10 times each for 5–8 s with 10 s intervals. Notably, the same point could not be stimulated twice in succession to avoid learning or sensitization. Data was expressed as the number of withdrawal responses of 10 applications, for which 0 indicated no withdrawal and 10 indicated the maximum number of withdrawals. Withdrawal responses were defined as (1) abdominal withdrawal from the VFFs, (2) consequent licking of the abdominal area, or (3) withdrawal of the entire body. All tests were performed in a blinded manner.

### Open field test (OFT)

The open field test (OFT) was performed to assess stress-induced anxiety-related psychiatric disorders [7]. The apparatus consisted of an open-square grey arena (40 × 40 cm), 40 cm high, with the floor divided into 16 squares by black lines. Each mouse was placed at the center of the open-field box, and its horizontal movements were monitored for 30 min with a video camera. Decreased time and distance in the central area were indicative of the psychiatric disorders-related behaviors. The apparatus was thoroughly cleaned between each test.

### Immunofluorescent staining

The ${\text{NA}}^{{{\text{LC}}}} \!\!\to\!\! {\text{RVM}}$ circuit was evaluated using immunofluorescent staining. The expression of c-Fos was examined to assess neuronal activity changes in the ${\text{NA}}^{{{\text{LC}}}} \!\!\to\!\! {\text{RVM}}$circuit after in vivo chemogenetic manipulation or DSS administration in hM3Dq-, hM4Di- and control mCherry-injected mice, with clozapine i.p. injection treatment for 2 h before perfusion [8, 31]. Mice were transcardially perfused with phosphate buffered saline (PBS) followed by 4% paraformaldehyde. The brain tissues were then harvested, fixed overnight in 4% paraformaldehyde at 4 °C, cryoprotected in 30% sucrose at 4 °C until isotonic and embedded in OCT. Free-floating immunohistochemistry was performed with 20-μm-thick serial coronal brain sections. After washing and blocking in blocking solution, the sections were incubated with primary antibodies, including both anti-c-Fos (1:500, rabbit, CST) and anti-TH (1:500, chk, Abcam) at 4 °C overnight, and with secondary antibodies (1:500, anti-rabbit Alexa Fluor 488, abcam and 1:500, anti-chk Alexa Fluor 405, abcam) at room temperature for 2 h. All antibodies were diluted in PBS prior to use. Confocal images were captured on an Olympus FV-1200 microscope.

### Histopathologic examination

After evaluating macroscopic damage, segments of the distal colon were stapled flat, mucosal side up, onto cardboard and fixed in 10% neutral-buffered formalin for 24 h at 4 °C. Samples were then dehydrated in sucrose, embedded in paraffin, sectioned at 5 μm and mounted onto slides. Paraffin blocks were prepared and sectioned at a thickness of 5 μm. Subsequently, the sections were transferred into hematoxylin and eosin and examined. Finally, photographs were taken with a digital imaging system consisting of a digital camera and image analysis software (Image J).

The microscopic assessments consisted of 3 items (severity of inflammation, mucosal damage, and crypt damage), and the highest score used for analysis. The score of each variable was added to give a total microscopic damage score (maximum of 11). Scores were defined as follows: goblet cell depletion score 1 (normal) or 0 (absence); crypt damage score 1 (normal) or 0 (absence); destruction of mucosal architecture score 1 (normal), 2 (moderate) or 3 (extensive); extent of muscle thickening score 1 (normal), 2 (moderate) or 3 (extensive); and presence and degree of cellular infiltration score 1 (normal), 2 (moderate) or 3 (transmural). The microscopic total damage score was determined in a blinded fashion. The resected sections of distal colon from the normal mice showed intact epithelium, normal muscle architecture and absence of edema, which are microscopic signs of colonic damage. Loss of mucosal architecture, thickening of smooth muscle, presence of crypt abscesses and extensive cellular infiltration were observed in the DSS-treated mice colon specimens.

### Statistical analysis

Statistical analyses were performed using GraphPad Prism 8 (GraphPad Software, San Diego, CA). Mice were randomly assigned to groups in all experiments. No animals or data points were excluded during analysis. The significance of the difference between two independent groups was determined using Student’s t tests. For multigroup comparisons, we applied analysis of variance (one-way or two-way ANOVA). The results were considered significantly different at *P* < 0.05. Data are presented as the mean ± SEM. Statistical details for specific experiments are summarized in the figure legends.

## Results

### The characteristics of the $\text{NA}^{\text{LC}} \!\!\to\!\! {\text{RVM}}$ circuit and facilitation of psychiatric disorders and visceral hyperalgesia after chemogenetic activation of the $\text{NA}^{\text{LC}} \!\!\to\!\! {\text{RVM}}$ circuit

**Fig. 1:**
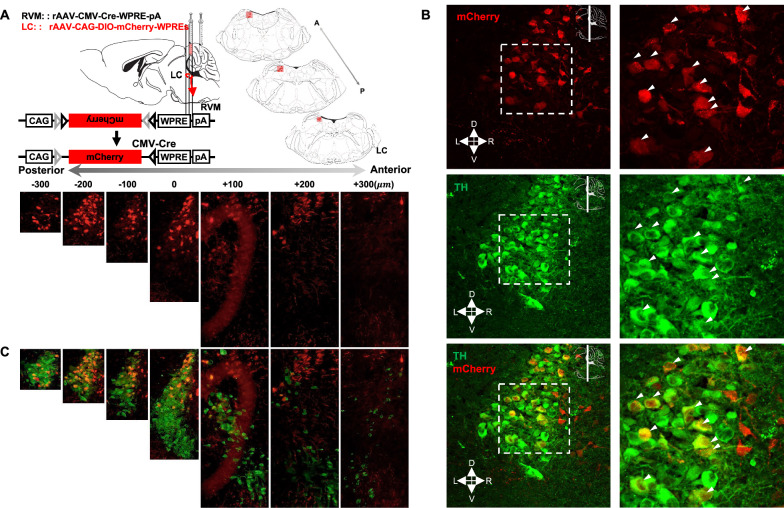
Dissection of the locus coeruleus noradrenergic neurons→rostral ventromedial medulla (NA^LC^→RVM) circuit. **A** Schematic diagram of injection and coronal sections of the LC. **B** Coronal section (at − 200 µm location) is shown. M: medial, D: dorsal, L: lateral, V: ventral. **C** mCherry (mCh) co-localizes with tyrosine hydroxylase (TH) in the LC derived from (**A**) and (**B**). Scale bars, 20 μm (**B**, Left), 50 μm (**B**, Right), 100 μm (**A**, Bottom and **C**)

To elucidate the precise location of LC neurons projecting to the RVM that could be involved in the development of visceral pain, we first investigated the distribution of mCherry signals in coronal sections of the entire LC after utilizing the adenovirus tracer for neuronal connectivity (Fig. 1A). Notably, many mCherry signals were observed in the dorsal caudal LC segment, which differed from the location of neurons in the LC that projected to the spinal cord. These data suggested that dorsal caudal LC neurons send afferents to the RVM.

We next identified the cell types of LC neurons projecting to the RVM. As the LC is the main source of noradrenaline in the central nervous system [55], we used immunohistochemical staining against tyrosine hydroxylase (TH) to localize LC-noradrenergic neurons (NA^LC^) (Fig. 1B) and marked the location of NA^LC^ projecting to the RVM (${\text{NA}}^{{{\text{LC}}}} \!\!\to\!\! {\text{RVM}}$) in each mouse (Fig. 1C). Approximately 96% of mCherry+ neurons in the LC were noradrenergic neurons.

**Fig. 2:**
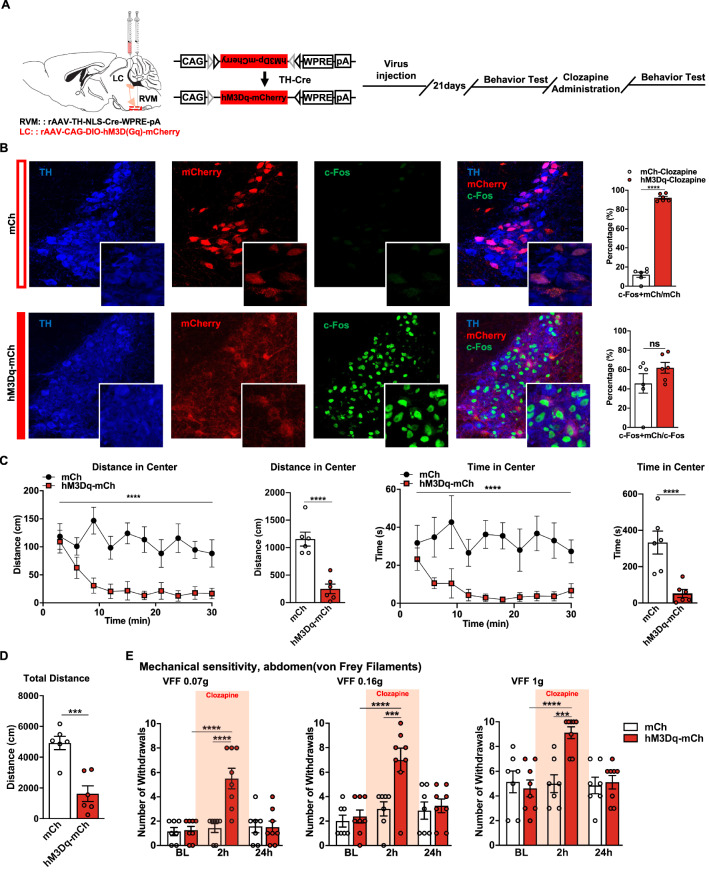
${\text{NA}}^{{{\text{LC}}}} \!\!\to\!\! {\text{RVM}}$ circuit activation drives anxiety-related psychiatric disorders and colorectal visceral hyperalgesia. **A** Schematic diagram of injection and timeline of experimental procedure. **B** (Left) Representative images of c-Fos expression in TH+/mCh+ neurons in the LC in hM3Dq-mCh and Control mice. (Right) The percentage of TH+/mCh+/c-Fos+ neurons in TH+/mCh+ or c-Fos+ neurons in hM3Dq-mCh and mCh mice 2 h after clozapine i.p. (*n* = 6 slices from three mice; c-Fos+mCh/mCh, *t* = 25.86, *P* < 0.0001; c-Fos+mCh/c-Fos, *t* = 1.406, *P* = 0.1899, unpaired t test). **C-D** Line charts and bars show the distance moved and the time spent in the center as well as the total distance moved immediately after clozapine i.p. in hM3Dq-mCh and mCh mice (*n* = 6 per group; distance in center: main effect: *F*_(1, 110)_ = 91.72, *P* < 0.0001, interaction: *F*_(9, 110)_ = 1.674, *P* = 0.1036; time in center: main effect: *F*_(1, 110)_ = 75.13, *P* < 0.0001, interaction: *F*_(9, 110)_ = 0.6825,* P* = 0.7233, two-way ANOVA with Sidak post hoc tests; total distance: *t* = 5.903,* P* = 0.0002, unpaired t test). **E** Mechanical sensitivity of the abdomen was measured with calibrated (0.07, 0.16, and 1 g) von Frey filaments (VFFs) before and 2 h and 24 h after clozapine i.p. in hM3Dq-mCh and mCh mice (*n* = 7–8 per group; 0.07 g, main effect: *F*_(1, 39)_ = 10.01, *P* = 0.0030, interaction: *F*_(2, 39)_ = 9.765, *P* < 0.0004; 0.16 g, main effect: *F*_(1, 39)_ = 8.472, *P* = 0.0059, interaction: *F*_(2, 39)_ = 4.873, *P* = 0.0129; 1.0 g, main effect:* F*_(1, 39)_ = 5.634, *P* = 0.0226, interaction: *F*_(2, 39)_ = 6.952, *P* = 0.0026, two-way ANOVA with Sidak post hoc tests). Scale bars, 20 μm (**B**, Right), 50 μm (**B**, Left). Data represent mean ± SEM. *P < 0.05, **P < 0.01, ***P < 0.001, ****P < 0.0001

To examine the role of the ${\text{NA}}^{{{\text{LC}}}} \!\!\to\!\! {\text{RVM}}$ circuit in psychiatric disorders and visceral hyperalgesia, we examined the effects of hM3D(Gq)-DREADD–mediated activation of this circuit in naïve mice (Fig. 2A). Neurons expressing the hM3Dq receptor can be activated by systemic clozapine administration (0.03 mg/kg i.p.). Immunostaining of c-Fos was carried out to evaluate the excitatory effect. As the results show, the expression of c-Fos remarkably increased after the administration of clozapine in the hM3Dq-mCh group compared with the mCh group. A total of 92.18% of the NA^LC^ projecting to the RVM were robustly activated (colocalized with c-Fos) compared with 11.99% in the mCh group (Fig. 2B).

Psychiatric disorders-related behaviors were evaluated by OFT immediately after clozapine injection, and the behavior of each mouse was recorded for 30 min. 5 min after administration of clozapine, hM3Dq-mCh mice displayed strongly suppressed locomotor activity and decreased distance moved. And they spent less time in the center of the open field (Fig. 2C, D).

Visceral pain-like hyperalgesia was assessed. Significant differences were found between the hM3Dq-mCh and the mCh groups. Clozapine increased abdominal responses in the hM3Dq-mCh group compared with the mCh group. By contrast, responses in the mCh group were not significantly different from the baseline (Fig. 2E). Thus, the activation of the ${\text{NA}}^{\text{LC}}\!\!\to\!\!{\text{RVM}}$ circuit could lead to anxiety-related psychiatric disorders and visceral hyperalgesia.

### Increased $\text{NA}^{\text{LC}} \!\!\to\!\! {\text{RVM}}$ circuit excitability in DSS-treated mice

**Fig. 3:**
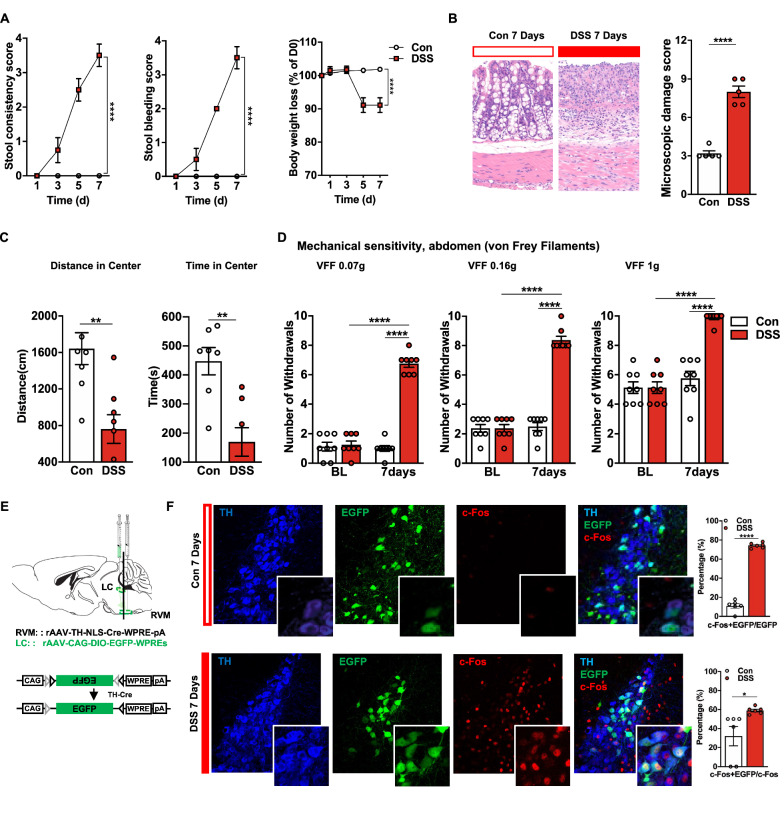
Increased activity of the ${\text{NA}}^{{{\text{LC}}}} \!\!\to\!\! {\text{RVM}}$ circuit in DSS-treated mice. **A** The stool consistency score (*n* = 8 per group, interaction: *F*_(3, 56)_= 29.38, *P* < 0.0001), the stool bleeding score (*n* = 8 per group, interaction: *F*_(3, 56)_ = 46.67, *P* < 0.0001) and the body weight loss (*n* = 8 per group, interaction: *F*_(4, 70)_=29.38, *P* < 0.0001) (two-way ANOVA with Sidak post hoc tests) were evaluated daily. **B** The microscopic damage score was a total score for 5 items (goblet cell depletion score; crypt damage score; destruction of mucosal architecture score; extent of muscle thickening score and presence and degree of cellular infiltration score) (maximum of 11) (*n* = 5 per group, *t* = 9.798, *P* < 0.0001, unpaired t test). **C** Bars show the distance moved and the time spent in the center (*n* = 8 per group; distance in center,* t* = 3.750, *P* = 0.0022; time in center, *t* = 4.077, *P* = 0.0015, unpaired t test). **D** Mechanical sensitivity of the abdomen was measured with calibrated (0.07, 0.16, and 1 g) VFFs in DSS and Control mice (*n* = 8 per group; 0.07 g, main effect: *F*_(1, 28)_ = 139.3, *P* < 0.0001, interaction:* F*_(1, 28)_ = 127.7, *P* < 0.0001; 0.16 g, main effect: *F*_(1, 28)_ = 123.7, *P* < 0.0001, interaction: *F*_(1, 28)_= 123.7, *P* < 0.0001; 1.0 g, main effect:* F*_(1, 28)_ = 29.66, *P* < 0.0001, interaction: *F*_(1, 28)_ = 29.66, *P* < 0.0001, two-way ANOVA with Sidak post hoc tests). **E** Schematic diagram of injection. **F** (Left) Representative images of c-Fos expression in TH+/EGFP+ neurons in the LC in DSS and Control mice. (Right) The percentage of TH+/EGFP+/c-Fos+ in TH+/EGFP+ or c-Fos+ neurons in DSS and control mice (*n* = 6 slices from 3 mice for each group; c-Fos + EGFP/EGFP,* t* = 23.66, *P* < 0.0001; c-Fos + EGFP/c-Fos,* t* = 2.624, *P* = 0.0254, unpaired t test). Scale bars, 20 μm (**F**, Left), 50 μm (**F**, Right). Data represent mean ± SEM. *P < 0.05, **P < 0.01, ****P < 0.0001

We used the DSS-induced colorectal visceral pain model to identify whether the $\text{NA}^{\text{LC}} \!\!\to\!\! {\text{RVM}}$ circuit is generally involved in pathological pain. Successful induction of the model was confirmed by increased stool consistency, appearance of blood in the stool, a marked reduction in body weight (Fig. 3A) and increased microscopic damage scoring of colon samples (Fig. 3B) 7 days after DSS administration compared with the control group.

The psychiatric disorders of mice were assessed after DSS treatment. In the OFT (Fig. 3C), DSS-treated mice showed significant decreases in center crossing and center duration compared with those in the control group. In addition, the impaired mobility among the DSS-treated mice could reflect visceral pain. Furthermore, the mean response frequencies of DSS-treated mice were significantly elevated compared to those of water-only treated mice at 7 days in the abdominal mechanical sensitivity evaluation (Fig. 3D). These results suggested that DSS administration successfully induced anxiety-related psychiatric disorders and colorectal visceral hyperalgesia.

To assess the neuronal excitability of the ${\text{NA}}^{{{\text{LC}}}} \!\!\to\!\! {\text{RVM}}$ circuit in visceral pain, we targeted noradrenergic neurons expressing TH by local injection of AAV2/9-CAG-DIO-EGFP into the LC after intra-RVM injection of AAV2/retro-TH-Cre to selectively highlight the NA^LC^→RVM circuit (Fig. 3E) and investigated the density of c-Fos-positive neurons coexpressing EGFP in the LC. We found that 74.36% of NA^LC^ projecting to the RVM were activated (colocalized with c-Fos) in the DSS group compared with 10.95% in the Con group (Fig. 3F). These results suggested an increase in neuronal excitability of the ${\text{NA}}^{{{\text{LC}}}} \!\!\to\!\! {\text{RVM}}$ circuit in DSS-induced colorectal visceral pain mice.

### Chemogenetic inhibition of the $\text{NA}^{\text{LC}} \!\!\to\!\! {\text{RVM}}$ circuit attenuates DSS-induced psychiatric disorders and visceral hyperalgesia

**Fig. 4:**
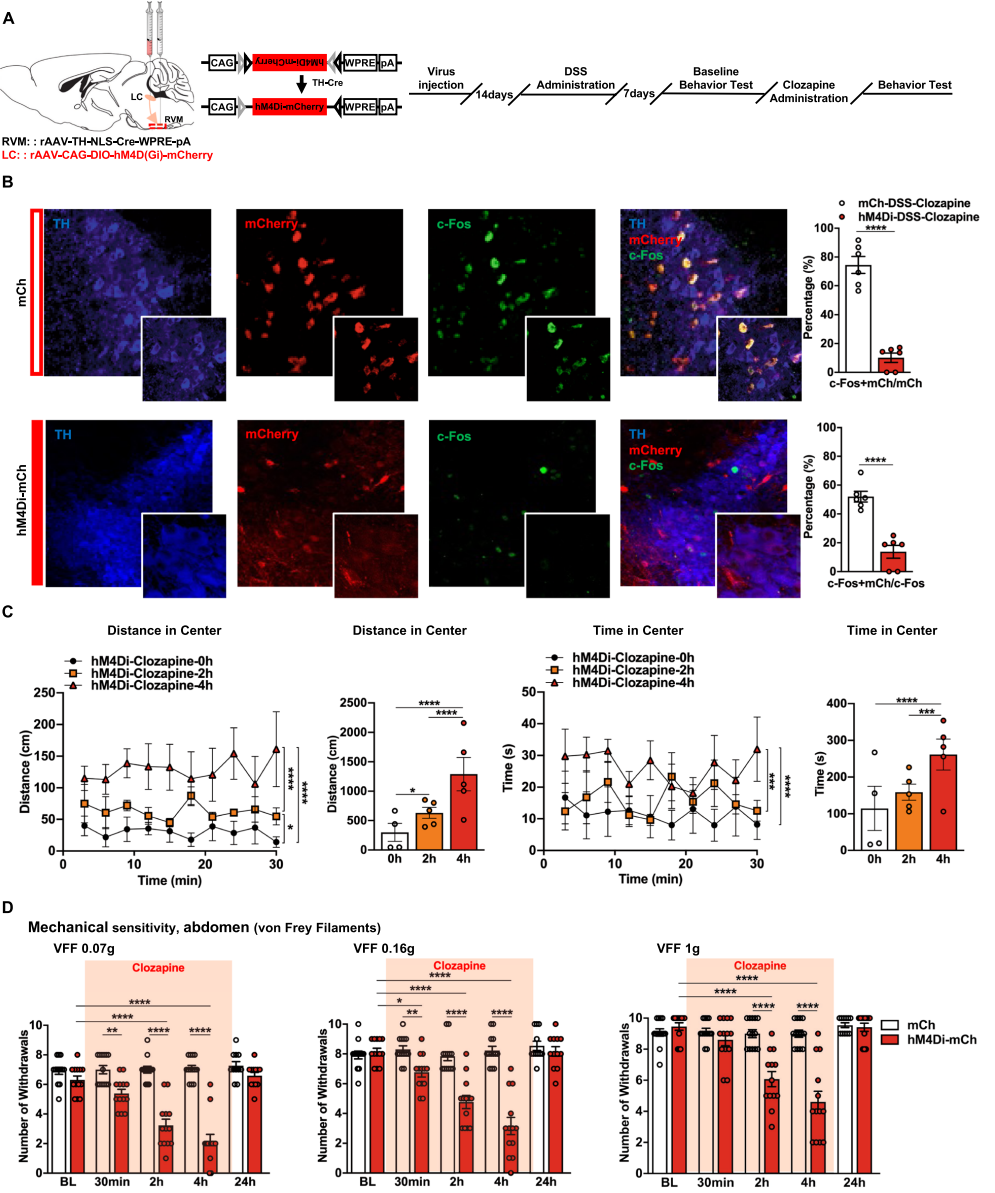
Decreasing the neuronal excitability of the ${\text{NA}}^{{{\text{LC}}}} \!\!\to\!\! {\text{RVM}}$ circuit attenuates anxiety-related psychiatric disorders and colorectal visceral hyperalgesia. **A** Schematic diagram of injection and timeline of experimental procedure. **B** (Left) Representative images of c-Fos expression in TH+/mCh+ neurons in the LC in DSS and control mice. (Right) The percentage of TH+/mCh+/c-Fos+ neurons in TH+/mCh+ or c-Fos+ neurons in hM4Di-mCh- and mCh-DSS mice 2 h after clozapine i.p. (*n* = 6 slices from three mice; c-Fos + mCh/mCh, *t* = 9.539, *P* < 0.0001; c-Fos + mCh/c-Fos, *t* = 6.609, *P* < 0.0001, unpaired t test). **C** Line charts and bars show the distance moved and the time spent in the center immediately, 2 h and 4 h after clozapine i.p. in hM4Di-mCh-DSS mice (*n* = 4–6 per group; distance in center, main effect: *F*_(2, 110)_=33.50,* P* < 0.0001; time in center, main effect: *F*_(2, 110)_ = 13.71, *P* < 0.0001, two-way ANOVA with Sidak post hoc tests). **D** Mechanical sensitivity of the abdomen was measured with calibrated (0.07, 0.16, and 1 g) VFFs before and 30 min, 2 h, 4 h and 24 h after clozapine i.p. in DSS mice expressing hM4Di-mCh or mCh (*n* = 13 per group; 0.07 g, main effect: *F*_(1, 117)_ = 146.1, *P* < 0.0001, time: *F*_(4, 117)_ = 21.08, *P* < 0.0001, interaction: *F*_(4, 117)_ = 20.32, *P* < 0.0001; 0.16 g, main effect of group: *F*_(1, 117)_= 80.57, *P* < 0.0001, time:* F*_(4, 117)_ = 21.60, *P* < 0.0001, interaction: *F*_(4, 117)_= 19.36, *P* < 0.0001; 1.0 g, main effect of group: *F*_(1, 117)_ = 45.76, *P* < 0.0001, time: *F*_(4, 117)_ = 21.59, *P* < 0.0001, interaction: *F*_(4, 117)_ = 16.78, *P* < 0.0001, two-way ANOVA with Sidak post hoc tests). Scale bars, 20 μm (**B**, Right) and 50 μm (**B**, Left). Data represent mean ± SEM. *P < 0.05, **P < 0.01, ***P < 0.001, ****P < 0.0001

**Fig. 5:**
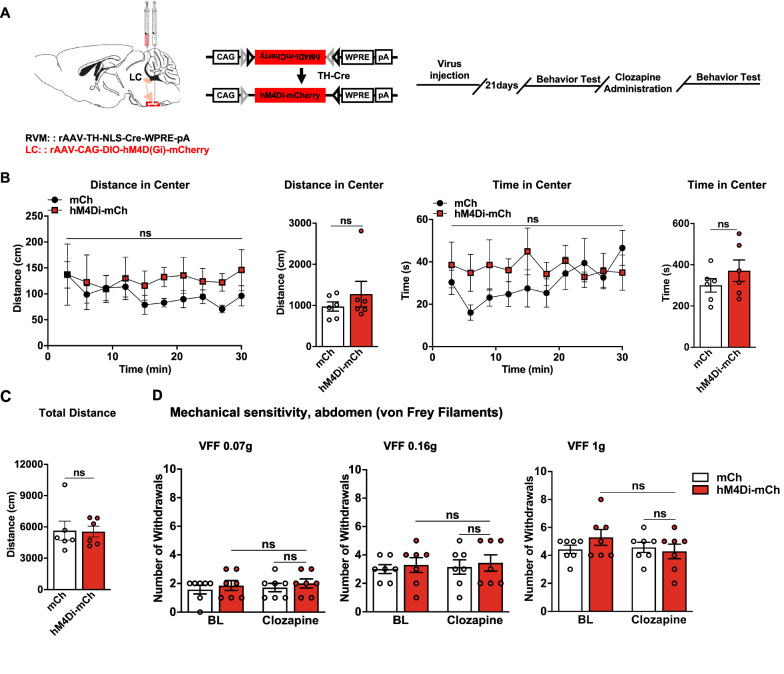
Decreasing the neuronal excitability of the ${\text{NA}}^{{{\text{LC}}}} \!\!\to\!\! {\text{RVM}}$ circuit has no effect on basal conditions. **A** Schematic diagram of injection and timeline of experimental procedure. **B** Bars show the distance moved and the time spent in the center as well as the total distance immediately after administration of clozapine (*n* = 6 per group; distance in center: main effect: *F*_(1, 100)_ = 5.488, *P* = 0.0211, interaction: *F*_(9, 100)_= 0.2461, *P* = 0.9865; time in center: main effect: *F*_(1, 100)_ = 4.131, *P* = 0.0448, interaction: *F*_(9, 100)_ = 0.8160, *P* = 0.6027, two-way ANOVA with Sidak post hoc tests). **C** Bars show the total distance moved after administration of clozapine i.p. in the hM4Di-mCh and mCh mice (*n* = 6 per group; total distance: *t* = 0.08644, *P* = 0.9328, unpaired t test). **D** Mechanical sensitivity of the abdomen was measured with calibrated (0.07, 0.16, and 1 g) VFFs before and 1 h after administration of clozapine in mice expressing hM4Di-mCh or mCh (*n* = 7 per group; 0.07 g, main effect: *F*_(1, 24)_ = 0.8571, *P* = 0.3638, interaction: *F*_(1, 24)_ = 0.000, *P* > 0.9999; 0.16 g, main effect: *F*_(1, 24)_ = 0.3429, *P* = 0.5637, interaction: *F*_(1, 24)_ = 3.313e-030, *P* > 0.9999; 1.0 g, main effect: *F*_(1, 24)_ = 0.4000, *P* = 0.5331, interaction: *F*_(1, 24)_ = 1.600, *P* = 0.2180 two-way ANOVA with Sidak post hoc tests). Data represent mean ± SEM. ns, no significance

Given the increased neuronal excitability of the ${\text{NA}}^{{{\text{LC}}}} \!\!\to\!\! {\text{RVM}}$ circuit in DSS-induced colorectal visceral pain mice, we subsequently aimed to investigate whether the inhibition of the neuronal excitability of the ${\text{NA}}^{{{\text{LC}}}} \!\!\to\!\! {\text{RVM}}$ circuit restored colorectal visceral ﻿hyperalgesia and anxiety-related psychiatric disorders in DSS-induced visceral pain mice (Fig. 4A). Neurons expressing hM4D(Gi) could be inhibited by systemic clozapine administration (0.1 mg/kg intraperitoneally). Immunostaining of c-Fos expression after 2 h of clozapine administration showed a dramatic decrease in the density of c-Fos-positive neurons coexpressing hM4Di-mCh in the LC compared with the mCh group. Only 10.16% of NA^LC^ projecting to the RVM were activated (colocalized with c-Fos) compared with 74.47% in the Con group (Fig. 4B). Psychiatric disorders-related behaviors were evaluated with 30 min recordings immediately and at 2 and 4 h after clozapine administration in the hM4Di-mCh-DSS group. The results showed that inhibiting ${\text{NA}}^{{{\text{LC}}}} \!\!\to\!\! {\text{RVM}}$ circuit induced a significant increase in the distance moved and the time spent in the center of the open field for the DSS group (Fig. 4C). Subsequently, colorectal visceral ﻿hyperalgesia-related responses markedly decreased in the hM4Di-mCh-DSS group at 2 and 4 h after clozapine administration. Responses in the DSS-mCh group were not significantly different from baseline at all assessed time points (Fig. 4D). Interestingly, in naïve mice, the inhibition of the ${\text{NA}}^{{{\text{LC}}}} \!\!\to\!\! {\text{RVM}}$ circuit has no affection on basal conditions based on locomotor and stress-related behaviors and pain threshold tests (Fig. 5A-C). Together, these findings indicate a crucial role of the ${\text{NA}}^{{{\text{LC}}}} \!\!\to\!\! {\text{RVM}}$ circuit in maintaining anxiety-related psychiatric disorders and colorectal visceral ﻿hyperalgesia in the current visceral pain model, while this circuit should be quiescent in the physiological state.

### Critical role of the $\text{NA}^{\text{LC}} \!\!\to\!\! {\text{RVM}}$ circuit in stress-induced colorectal visceral hyperalgesia

**Fig. 6:**
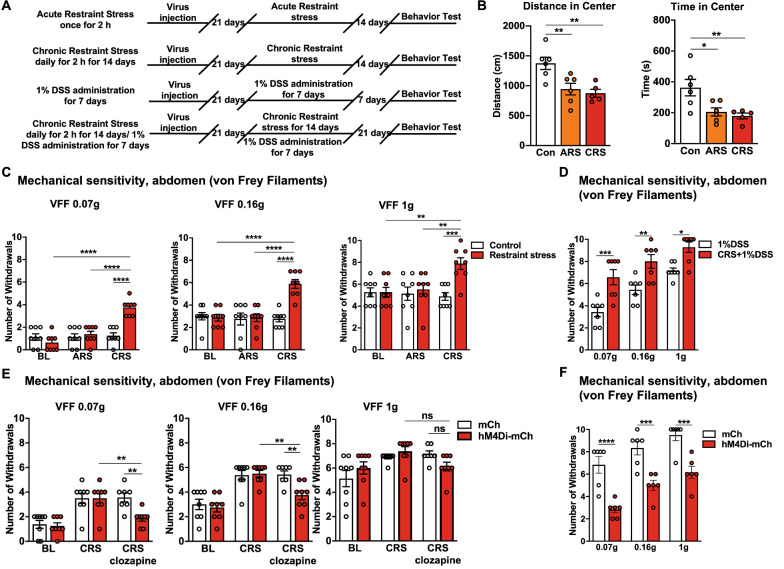
Decreasing the neuronal excitability of the ${\text{NA}}^{{{\text{LC}}}} \!\!\to\!\! {\text{RVM}}$ circuit attenuates CRS-induced anxiety-related psychiatric disorders and colorectal visceral hyperalgesia. **A** Timeline of experimental procedure. **B** Bars show the distance moved and the time spent in the center in the ARS, CRS, and control mice (*n* = 6 per group; distance in center,*F*_(2,14)_ = 8.401, *P* = 0.0040; time in center, *F*_(2,14)_ = 6.946, *P* = 0.0080, one-way ANOVA with Dunnett post hoc analysis). **C** Mechanical sensitivity of the abdomen was measured in the ARS, the CRS and the control mice (*n* = 8 per group; 0.07 g, main effect: *F*_(1,42)_ = 11.57, *P* = 0.0015, interaction: *F*_(2,42)_= 16.71, *P* < 0.0001; 0.16 g, main effect: *F*_(1,42)_= 11.99, *P* = 0.0012, interaction: *F*_(2,42)_ = 12.04, *P* < 0.0001; 1.0 g, main effect of group: *F*_(1,42)_= 7.628, *P* = 0.0085, interaction: *F*_(2,42)_= 5.368,*P* = 0.0084, two-way ANOVA with Tukey post hoc tests). **D** Mechanical sensitivity of the abdomen was measured in the 1% DSS and CRS + 1% DSS mice (*n* = 7 per group, main effect: *F*_(1,36)_ = 40.88, *P* < 0.0001, interaction: *F*_(2,36)_ = 0.50, *P* = 0.6107, two-way ANOVA with Sidak post hoc tests). **E** Mechanical sensitivity of the abdomen was measured before and 2 h after clozapine i.p. in control and hM4Di-mCh- or mCh-CRS mice (*n* = 7–8 per group; 0.07 g, main effect: *F*_(1,41)_ = 4.666, *P* = 0.0367, interaction: *F*_(2,41)_ = 3.686, *P* < 0.0337; 0.16 g, main effect: *F*_(1,41)_ = 4.279, *P* = 0.0449, interaction: *F *_(2,41)_ = 3.500, *P* = 0.0395; VFFs 1.0 g, main effect: *F*_(1,41)_ = 0.1187,*P* = 0.7322, interaction: *F*_(2,41)_ = 2.746, *P* = 0.0760, two-way ANOVA with Tukey post hoc tests). **F** Mechanical sensitivity of the abdomen was measured before and 2 h after clozapine i.p. in hM4Di-mCh- or mCh-CRS + 1% DSS mice (*n* = 6 per group, main effect: *F*_(1,30)_= 64.00, *P* < 0.0001, interaction: * F*_(2,30)_ = 0.25, *P* = 0.7804, two-way ANOVA with Sidak post hoc tests). Data represent mean ± SEM. *P < 0.05, **P < 0.01, ***P < 0.001, ****P < 0.0001

**Fig. 7:**
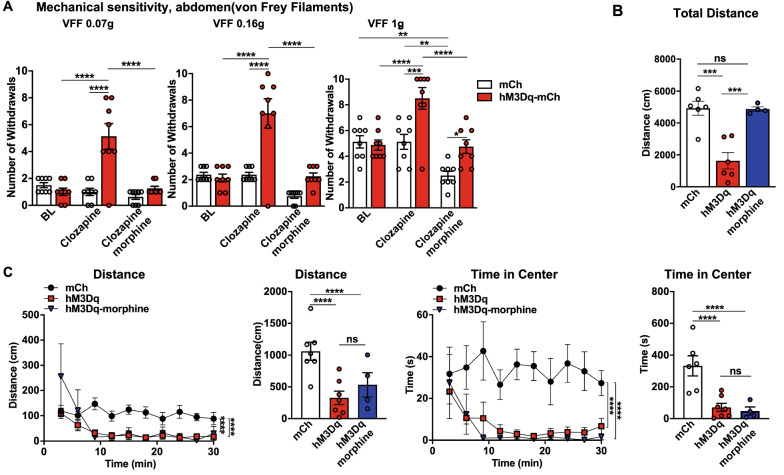
The ${\text{NA}}^{{{\text{LC}}}} \!\!\to\!\! {\text{RVM}}$ circuit controls stress-induced colorectal visceral hyperalgesia. **A** Mechanical sensitivity of the abdomen was measured with calibrated (0.07, 0.16, and 1 g) VFFs before and 2 h after clozapine i.p. and 30 min after morphine i.p. which was followed by clozapine by 90 min i.p. in hM3Dq-mCh or mCh mice (*n* = 8 per group; 0.07 g, main effect: *F*_(1,42)_ = 15.74, *P* = 0.0003, interaction: *F*_(2,42)_ = 15.21, *P* < 0.0001; VFFs 0.16 g, main effect: *F*_(1,42)_ = 23.68,*P* < 0.0001, interaction: *F*_(2,42)_ = 12.55, *P* < 0.0001; VFFs 1.0 g, main effect: *F*_(1,42)_ = 15.84,*P* = 0.0003, interaction: *F*_(2,42)_ = 5.663, *P* = 0.0066, two-way ANOVA with Sidak post hoc tests). **B-C** Bars show the distance moved, the time spent in the center and the total distance moved immediately after clozapine i.p. followed after 30 min by morphine i.p. (*n* = 6 per group; total distance: *F*_(2,15)_ = 8.416, *P* = 0.0035, one-way ANOVA with Dunnett post hoc analysis; distance in center: main effect: *F*_(2,140)_ = 25.44,*P* < 0.0001, interaction: *F*_(18,140)_ = 2.028,*P* = 0.0118; time in center: main effect: *F*_(2,140)_ = 53.61, *P* < 0.0001, interaction: *F*_(18,140)_ = 0.6189, *P* = 0.8802, two-way ANOVA with Tukey post hoc tests). Data represent mean ± SEM. *P < 0.05, **P < 0.01, ***P < 0.001, ****P < 0.0001

Based on the previous results, we subsequently investigated the specific involvement of the ${\text{NA}}^{{{\text{LC}}}} \!\!\to\!\! {\text{RVM}}$ circuit in stress-induced colorectal visceral ﻿hyperalgesia. The CRS model was used to induce anxiety-related psychiatric disorders and the CRS combined with 1% DSS model was used to replicate the common condition that psychiatric disorders predispose the individual to develop visceral hyperalgesia (Fig. 6A). In the OFT, the mice in both groups displayed significant anxiety-related psychiatric disorders compared with the control group (Fig. 6B). Interestingly, colorectal visceral hyperalgesia-related behavior was also observed in the CRS group, while it was absent in the control and ARS groups (Fig. 6C and D). These results suggested that the current CRS and CRS combined with 1% DSS treatment models reliably induced colorectal visceral hyperalgesia behavior. We next investigated whether the inhibition of the neuronal excitability of the ${\text{NA}}^{{{\text{LC}}}} \!\!\to\!\! {\text{RVM}}$ circuit restored stress-induced colorectal visceral hyperalgesia. The chemogenetic inhibition of the ${\text{NA}}^{{{\text{LC}}}} \!\!\to\!\! {\text{RVM}}$ circuit did decrease colorectal visceral hyperalgesia-related responses in the CRS and CRS combined with 1% DSS treatment mice (Fig. 6E and F). In addition, activation of the ${\text{NA}}^{{{\text{LC}}}} \!\!\to\!\! {\text{RVM}}$ circuit produces anxiety-related psychiatric disorders and colorectal visceral hyperalgesia, but acutely inhibiting colorectal visceral hyperalgesia with morphine (0.2% 0.1 ml/10 g i.p.) had no anti-psychiatric disorders effects. We found that the movement distance and time spent at the center of the open field were similar, but the total distance of locomotor activity increased in the hM3Dq-mCh-clozapine-morphine group compared with the hM3Dq-mCh-clozapine group (Fig. 7A–C). These results suggested a crucial role of the ${\text{NA}}^{{{\text{LC}}}} \!\!\to\!\! {\text{RVM}}$ circuit in the development of stress-induced colorectal visceral hyperalgesia instead of colorectal visceral pain-facilitated psychiatric disorders.

## Discussion

Our study demonstrated a previously unappreciated ${\text{NA}}^{{{\text{LC}}}} \!\!\to\!\! {\text{RVM}}$ circuit that mediates stress-induced colorectal visceral hyperalgesia. Specifically, stress could activate LC noradrenergic neurons that project to the RVM and induced enhanced visceral pain sensation via the RVM-related descending pain facilitation pathway. Inhibiting this circuit effectively alleviates stress-induced colorectal visceral pain.

Accumulating evidences has demonstrated that LC neurons contribute to widespread projection networks throughout the central nervous system, and well-defined subpopulations of these neurons can encode distinct processes [52, 58, 70]. Therefore, the LC is assumed to be involved in both pain facilitation and alleviation [2, 19, 67]. Previously, visceral nociceptive responses of spinal dorsal horn neurons were reported to be inhibited by the descending LC-spinal circuit in the rat [27, 38]. Thus, the LC may exert a pro-nociceptive effect via a different circuit. Specifically, the activation of ${\text{NA}}^{{{\text{LC}}}} \!\!\to\!\! {\text{RVM}}$ projections found in this study facilitates colorectal visceral pain. The RVM has been recognized as a pain-gating nucleus responsible for integration of pain inhibition and facilitation processing since the early 1980s [56, 71, 72]. Although noradrenergic input to RVM nociceptive modulatory neurons has been reported [9, 46, 66] and interactions between the LC and RVM have been proven via fMRI experiments, the precise role of this projection in pain modulation has never been studied. Our results suggest that LC-noradrenergic neurons send monosynaptic projections to the RVM and that the selective chemogenetic activation of LC neurons that project to the RVM elicits abdominal hyperalgesia in naïve mice. Considering these findings, we propose that the LC exerts an indirect pro-nociceptive effect of visceral pain due to its projections to the RVM. Indeed, unlike the visceral pain inhibition effect of known LC-spinal cord descending projections, the LC-RVM circuit in this study acts to promote visceral pain, which is consistent with the bidirectionally influence of the LC in pain modulation [24]. In addition, understanding the anatomical organization of the LC is critical to unlocking its function [52]. Counter to previous reports that showed that antinociceptive actions were evoked from a distinct, ventral subpopulation of LC neurons [24], we found that LC neurons projecting to the RVM were predominantly located in the dorsal part of the LC. This finding is aligned with the heterogeneity of LC and demonstrates the bidirectional regulation of nociception by LC [58].

Frequently, pathological stress tends to enhance visceral pain, coinciding with clinical observation [4, 17, 34], and the LC is a crucial component in both stress- and pain-related neural circuits [24, 32, 44, 69]. However, limited data elucidate the endogenous circuit mechanisms underlying stress and visceral pain interactions associated with the LC. The LC has only been thought to form part of a descending endogenous analgesic system that exerts inhibitory influences on spinal nociception [75]. The increased inhibition of the LC from the amygdala was once seen as a potential pain-enhancing mechanism for emotions processed in peripheral neuropathy [74]. Notably, this circuit does not participate in the promotion of emotion-induced visceral pain facilitation [74]. Theoretically, the heterogeneity of the LC and bidirectional modulation of nociception by the LC leads to multifaceted effects on visceral pain through distinct neural circuits. In this study, DSS-induced colorectal visceral pain in mice lowered sensory thresholds and provoked a stress-related profile; meanwhile, the ${\text{NA}}^{{{\text{LC}}}} \!\!\to\!\! {\text{RVM}}$ circuit was significantly activated, as shown by significantly increased numbers of c-Fos-positive neurons in the LC. Interestingly, the chemogenetic activation of the ${\text{NA}}^{{{\text{LC}}}} \!\!\to\!\! {\text{RVM}}$ circuit in naïve mice instantly induced stress-related psychiatric disorders and subsequently, visceral hyperalgesia. In contrast, chemogenetic inhibition of the ${\text{NA}}^{{{\text{LC}}}} \!\!\to\!\! {\text{RVM}}$ circuit attenuates psychological maladaptation and pathological visceral hyperalgesia in the CRS and DSS models without affecting physiological pain sensation. Moreover, opioids are the frontline analgesics in pain management, and morphine is a potent opioid analgesic. The microinjection of morphine into the LC was shown to elicit analgesia through LC-Spinal cord projections, and it did not modify pain aversion [40]. Acute pain inhibition with morphine does not reverse the activity of the ${\text{NA}}^{{{\text{LC}}}} \!\!\to\!\! {\text{RVM}}$ circuit and does not attenuate psychiatric disorders-induced by the activation of this circuit. Therefore, these results seem to indicate that modulatin the ${\text{NA}}^{{{\text{LC}}}} \!\!\to\!\! {\text{RVM}}$ circuit affected both stress-related psychiatric disorders and pain sensory perception in the current animal models.

Earlier studies focused on the antinociceptive effects of RVM by exerting descending inhibitory effects on the spinal cord, but more recently, the RVM was shown to promote spinal pain signaling and increase nociceptive sensitivity. Multiple studies have also shown that RVM neurons play an important role in visceral hyperalgesia [54, 78], and noradrenergic inputs have been demonstrated to affect pain modulation by RVM neurons [5, 23]. In addition, we have demonstrated that activating the RVM receiving direct input from the NA^LC^ can enhance colorectal visceral pain perception in case of visceral inflammation-induced physical stress or restraint-induced psychological stress. Therefore, the LC can reasonably be assumed to be involved in stress-induced colorectal visceral pain via RVM. Noradrenaline plays a role in the modulation of pain in the RVM via α1- and α2-adrenoceptors on neurons. Previous studies have indicated that microinjecting α1 agonists and α2 agonists into the RVM increases and decreases nociceptive responses, respectively [26, 53]. Thus, the increased activation of α1-adrenoceptors from the LC on RVM neurons might cause an induced visceral pain-enhancing effect in mice with visceral inflammation or psychology-induced stress. However, the RVM has functionally distinct cell groups, and the cellular distribution and synaptic actions of α-receptor subtypes in the different cell groups are unknown. Thus, whether and how α receptors is involved in this circuit needs further investigation.

This study certainly has some important limitations. We did not address why chemogenetic manipulation of the ${\text{NA}}^{{{\text{LC}}}} \!\!\to\!\! {\text{RVM}}$ affects psychological maladaptation in this study, but we are very interested in this topic. The locus coeruleus (LC)-noradrenergic system is the main source of noradrenaline in the central nervous system and is intensively involved in modulating stress-related psychiatric disorders (major depressive disorder and anxiety) [45, 64]. When evaluating the chemogenetic activation of the LC, ongoing behaviors are rapidly interrupted and exploratory activity and anxiety are increased [25]. The chemogenetic blockade of LC neurons projecting to the ACC completely reverses depressive-like behavior [39]. Therefore, we cannot conclude that the ${\text{NA}}^{{{\text{LC}}}} \!\!\to\!\! {\text{RVM}}$ circuit is the only mechanism by which the LC modulates stress-psychiatric disorders, although the activation of this circuit is well known to instantly induce a robust suppression of locomotion and exploration. Indeed, we observed a strong increase in the number of c-Fos-positive NA^LC^ neurons, but this increase was not restricted to mCh coexpression in hM3Dq-mCh mice. The neuronal responses we observed may be mediated by local or distal polysynaptic recurrent circuits. In fact, our chemogenetic strategy specifically activates LC neurons and rapidly induces stress-related psychiatric disorders changes that last at least 30 min. Therefore, an increase in the activity of LC neurons projections to RVM activity might rapidly reconfigure large-scale brain networks to send projections to structure involved in emotions, which contribute to psychiatric disorders. Future investigations are needed to elucidate the involvement of this circuit in local or distal polysynaptic recurrent circuits. In addition, we mainly focused on visceral hyperalgesia, and a single OFT test was applied to assess psychiatric disorders. However, additional experiments (such as the plus-maze test and tail suspension test) could be helpful [11, 14] and will be included in a follow-up paper.

Overall, in addition to stress-related psychiatric disorders, our study extends LC function to colorectal visceral pain modulation through descending projections to the RVM, which are distinct from the LC/SC projections. The reorganization of the LC functional structure in the presence of stress will lead to activation of the ${\text{NA}}^{{{\text{LC}}}} \!\!\to\!\! {\text{RVM}}$ circuit to facilitate colorectal visceral pain, while the inhibition of this circuit can robustly alleviate stress-induced colorectal visceral pain. The LC likely acts as a relay center, exchanging information on stress-related psychiatric disorders and visceral pain. These findings provide a new perspective for exploring stress-induced pain sensation changes in neural circuits and raise the possibility of a novel therapeutic target for the management of stress-induced colorectal visceral hyperalgesia.

## Data Availability

All data generated or analysed during this study are included in this published article [and its supplementary information files].

## Abbreviations

AAVs: Adeno-associated viruses
ARS: Acute restraint stress
AWR: Abdominal withdrawal reflex
CRS: Chronic restraint stress
DREADD: Designer receptors exclusively activated by designer drugs
DSS: Dextran sulfate sodium
EGFP: An enhanced green fluorescent protein
hM3Dq: Human M3 muscarinic DREADD receptor coupled to Gq
hM4Di: Human M4 muscarinic DREADD receptor coupled to Gi
LC: Locus coeruleus
LC/SC: Coeruleospinal
mCh: mCherry fluorescent protein
NA: Noradrenaline
NA^LC^: LC-noradrenergic neurons
NA^LC^→RVM: The NA^LC^ projecting to the RVM
OFT: Open field test
RVM: Rostral ventromedial medulla
TH: Tyrosine hydroxylase
VFFs: Von frey filaments
